# A novel nowcasting (estimation) model based on an adaptive network neutrosophic hesitant fuzzy inference system (ANNHFIS): a case study of Istanbul

**DOI:** 10.1038/s41598-026-45618-7

**Published:** 2026-03-24

**Authors:** Ataullah Turgut, Sukran Seker

**Affiliations:** Department of Industrial Engineering, Yildiz Technical University, Istanbul, 34349 Turkey

**Keywords:** Adaptive neuro-fuzzy inference system (ANFIS), Air pollution, Biomass power plants, Neutrosophic hesitant fuzzy sets (NHFS), Nitrogen dioxide (NO₂) estimation, Particle swarm optimization (PSO), Energy science and technology, Engineering, Mathematics and computing

## Abstract

Although biomass power plants are cleaner than fossil-fuel-based plants, they emit nitrogen dioxide (NO₂), which can degrade urban air quality and pose respiratory health risks. Therefore, reliable estimation (nowcasting) of NO₂ levels around these facilities is crucial for public health and air quality management. This study proposes an adaptive network-based neutrosophic hesitant fuzzy inference system optimized by particle swarm optimization (ANNHFIS-PSO) to estimate NO₂ concentrations near biomass plants in Istanbul. To our knowledge, this is the first adaptive neuro-fuzzy inference system (ANFIS)-based framework that incorporates neutrosophic hesitant fuzzy sets to represent environmental uncertainty. The proposed model integrates a neural network with neutrosophic hesitant fuzzy membership functions and employs a hybrid learning scheme that combines PSO-based global optimization with Adam-based fine-tuning to capture nonlinear relationships. Its performance was benchmarked against multilayer perceptron artificial neural network (MLP-ANN), ANFIS-PSO, grid-search-tuned ANFIS (ANFIS-GS), long short-term memory (LSTM) network and ANNHFIS-GS. Model accuracy was evaluated using metrics including root mean square error (RMSE) and coefficient of determination (R²). On the test dataset, ANNHFIS-PSO achieved an RMSE of 3.6488 µg/m³ and an R² of 0.8938, yielding the lowest RMSE and a high R² among the evaluated models. These results suggest that the proposed approach may support decision-making for air quality management near biomass plants.

**Supplementary Information:**

The online version contains supplementary material available at 10.1038/s41598-026-45618-7.

## Introduction

Air pollution comprises a complex blend of solid particles, liquid droplets, and gaseous substances and it originates from various sources, such as the combustion of household fuels, emissions from industrial chimneys, vehicle exhaust, electricity generation, the open burning of waste, agricultural activities and desert dust. The World Health Organization (WHO) has established air pollutant limits to safeguard public health. Accordingly, to improve air quality, the WHO guidelines recommend limits and interim targets for common air pollutants, including particulate matter (PM), ozone (O₃), nitrogen dioxide (NO₂), and sulfur dioxide (SO₂)^1^.

While nitrogen oxides (NOₓ) comprise various chemical species, NO₂ is the most significant air pollutant affecting human health^2^. NO₂ plays a critical role in shaping the functioning of the atmosphere because of its effects on both human health and ecological systems. Although NO₂ absorbs sunlight, it also limits air clarity and contributes to climate change^3,4^. NO₂ drives atmospheric reactions that form nitric and sulfuric acids and, under solar radiation, generate organic, nitrate and sulfate aerosols classified as inhalable PM (PM₁₀) and fine PM (PM₂․₅). It is therefore a major precursor of secondary pollutants and is associated with adverse respiratory effects in humans^5^.

Biomass power plants, which are regarded as a cleaner alternative to conventional fossil fuel-based power generation, convert organic waste into energy and supply electricity. However, depending on the type of fuel used, the combustion conditions and the pollution control measures in place, biomass combustion can lead to the emission of certain pollutants, including PM and NOₓ, with emission levels that are comparable to those from fossil fuels. This necessitates a cautious evaluation of the classification of biomass as a clean energy source^6^.

Given these environmental and public health concerns, accurate predictions of air pollutant concentrations have become an important research focus. Recent work has also addressed street-level NO₂ nowcasting using Gaussian-process-based models that exploit spatial correlations from dense sensor networks and provide predictive uncertainty (e.g., via the associated standard deviation)^7^. In the literature, air pollution prediction studies have focused primarily on PM₂.₅ and PM₁₀^8-14^ while also addressing other pollutants, such as carbon monoxide (CO)^15^, NOₓ, NO₂^16^, SO₂ and O₃^17^, alongside forecasting the air quality index (AQI)^18–20^.

In these studies, meteorological parameters such as temperature, relative humidity, precipitation and wind are generally used as model inputs, while the target pollutant is selected as the model output. In some cases, to increase the prediction accuracy, additional pollutants other than the target pollutant are included as input variables together with meteorological parameters^18,21–23^.

To predict the air quality, both linear and nonlinear model structures have been used in the literature. Linear approaches—including autoregressive moving average with exogenous inputs (ARMAX), autoregressive with exogenous inputs (ARX), Box–Jenkins and output–error models—have been applied to predict PM₁₀ pollution and have achieved successful results^9^. In addition to autoregressive integrated moving average (ARIMA) models, which combine linear and nonlinear components, hybrid schemes such as generalized autoregressive conditional heteroskedasticity (GARCH)-ARIMA and wavelet transform (WT)-ARIMA models have been employed to enhance air pollutant prediction performance^8,12^.

Nevertheless, air pollution modelling is inherently complex and involves nonlinear behaviour and multiple interacting components^17^. Because conventional time series approaches assume linear relationships among variables, statistical models cannot adequately represent complex patterns in nonlinear time series and therefore exhibit limited suitability, particularly for long-term forecasting^24^.

To overcome these limitations, machine learning-based methods have been extensively adopted in recent years. In this context, deep learning models, particularly long short-term memory (LSTM) networks, have demonstrated a notable ability to model nonlinear relationships. Furthermore, recent comparative studies evaluating advanced deep-learning architectures for NO₂ prediction report that Transformer-based time-series models achieved the best performance across multiple monitoring stations relative to several baselines, including LSTM models^25^. By employing particle swarm optimization (PSO) to optimize hyperparameters and avoid random selection, better results have been obtained than those of traditional methods such as ARIMA models and radial basis function neural networks (RBFNNs)^26–29^.

Machine learning approaches, especially artificial neural network (ANN)-based models, have emerged as effective and increasingly popular alternatives to traditional techniques for modelling air pollutants^30–32^. In a comparative study conducted to predict urban NO₂ and PM₁₀ concentrations, five distinct ANN architectures, one linear statistical model and one deterministic modelling system were evaluated; the results revealed that the ANNs outperformed the other approaches in terms of prediction accuracy^33^. However, ANN-based methods may not always achieve the expected level of accuracy. Convergence to local minima during optimization and a tendency to overfit the training data are among the main limitations of these methods^34^.

The adaptive neuro-fuzzy inference system (ANFIS) is another effective approach for modelling complex nonlinear relationships^17,30,35^. In a study aiming to compare semiempirical nonlinear regression and ANFIS models for predicting the concentrations of four major pollutants (CO, SO₂, O₃ and NO₂), the ANFIS model was reported to capture the nonlinear nature of air pollution more reliably^17^. Additionally, ANFIS frameworks have been applied to spatiotemporal air-pollution modelling; for example, by integrating satellite-based observations with traffic, meteorological, and land-use predictors, ANFIS was shown to capture the spatiotemporal variability of monthly mean NO₂ concentrations at 1-km spatial resolution^36^. Moreover, reducing redundant inputs has been highlighted in ANFIS-based air-quality forecasting; collinearity-based screening combined with stepwise forward selection can reduce the number of candidate input subsets, thereby lowering computational cost and time while maintaining predictive accuracy^22^.

In ANFIS-based forecasting, the selection of the initial population strongly affects convergence behaviour towards the optimal solution; therefore, various hybrid models that exploit metaheuristic algorithms have been developed. For instance, recent studies have trained ANFIS using popular metaheuristic algorithms such as GA, PSO, and differential evolution (DE) for air pollution modelling in Istanbul, reporting that metaheuristic-trained ANFIS configurations yielded more accurate predictions than the classical ANFIS model^37^. In a broader comparison regarding the prediction of PM₂.₅, SO₂ and NO₂, ANFIS models optimized by the PSO algorithm, the genetic algorithm (GA), the slime-mould algorithm (SMA), the sine cosine algorithm (SCA), the salt swarm algorithm (SSA) and a PSO-SMA hybrid algorithm were evaluated. The hybrid PSO-SMA ANFIS model outperformed both the hybrid and standalone ANFIS models, whereas the SCA- and SSA-based hybrid ANFIS models performed worse than the standalone ANFIS configurations^38^. These findings highlight that the choice of an appropriate metaheuristic optimization scheme is critical in ANFIS-based forecasting systems.

However, classical neuro-fuzzy models that rely on a single membership function cannot adequately represent both membership and nonmembership states. Moreover, factors beyond error and probability distributions introduce nonstochastic uncertainties into the selection of the membership function^39^. To explicitly represent indeterminacy, neutrosophic-set-based extensions of ANFIS have also been proposed; for instance, crisp inputs can be transformed into single-valued neutrosophic numbers via triangular/trapezoidal neutrosophic membership functions and then processed through an ANFIS framework^40^. Nevertheless, to address these limitations, a deterministic and probabilistic air quality forecasting system based on hesitant fuzzy sets with nonlinear error correction, which also accounts for outliers, has been proposed; this system has been reported to provide more accurate and reliable results than alternative methods^41^. Furthermore, the synergy between neutrosophic logic and hesitant fuzzy sets has been explored for time-series forecasting; specifically, a modified single-valued neutrosophic hesitant fuzzy time series model that incorporates multiple hesitancy degrees via Gaussian and bell-shaped membership functions was reported to improve forecasting accuracy compared with benchmark approaches such as Sugeno-ANFIS, ARIMA and LSTM^42^. Collectively, these studies indicate a need for new hybrid model designs that can represent uncertainty more richly and are supported by advanced optimization algorithms for air pollution prediction.

Motivated by this gap, to the best of our knowledge, integrating neutrosophic hesitant fuzzy sets (NHFS) into the ANFIS architecture for same-day air-quality nowcasting has not been explicitly investigated. This study addresses this gap by proposing an adaptive network-based neutrosophic hesitant fuzzy inference system optimized by particle swarm optimization (ANNHFIS-PSO) model, which combines the adaptive learning capability of ANFIS with the uncertainty-modeling capabilities of NHFS. The main contributions of this work are as follows:

- First, integrating NHFS aims to provide a structured mechanism to capture uncertainty and hesitation in the input space.
- Second, a Hamacher T-norm-based aggregation operator is employed to achieve smoother gradient transitions in overlapping fuzzy regions.
- Third, a hybrid learning scheme is introduced, in which PSO globally optimizes the premise (membership-function) parameters and the rule-weight coefficients, while the consequent parameters are estimated via ridge-regularized least squares at each epoch. The best solution is then refined using Adam-based fine-tuning to enhance local convergence and generalization.

In the following sections, we first describe the study area and datasets, then present the proposed ANNHFIS-PSO model, report the comparative results and finally discuss the key findings and conclusions.

## Materials and methods

### Study area and available data

Istanbul is the most populous city in Türkiye, with approximately 16 million inhabitants, corresponding to approximately 19% of the national population^43^. Municipal solid waste management, street cleaning and recycling services are coordinated by İSTAÇ A.Ş., a subsidiary of the Istanbul Metropolitan Municipality^44^. İSTAÇ operates integrated waste-to-energy and biomethanization facilities that simultaneously manage municipal waste disposal and electricity generation^45^.

In 2023, these biomass power plants generated approximately 1.3 × 10⁶ MWh of electricity. This amount is sufficient to meet the electricity demand of approximately 2.5 million residents in Istanbul. The contributions of the Eyüp, Silivri and Şile districts to biomass-based electricity production are approximately 58%, 18% and 23% of the total generation, respectively^46^.

Because Eyüp has the highest waste-to-energy capacity, it was selected as the study area. In 2023, a total of 1,364,169 tons of municipal solid waste were processed at the waste-to-energy and biomethanization plants located in Eyüp^46^. While this large-scale waste processing supports renewable electricity generation, it also leads to the emission of major air pollutants such as carbon dioxide (CO₂), NO₂, SO₂ and PM₂.₅ into the atmosphere.

In the proposed estimation (nowcasting) model, the following five variables were used as inputs: solar radiation (W/m²), air temperature (°C), relative humidity (%), NO_x_ (µg/m³) and PM₁₀ (µg/m³). NO₂ (µg/m³) was considered the output variable. These predictors were selected because solar radiation controls photochemical reactions, air temperature and relative humidity influence the dispersion and transformation of pollutants in the atmosphere, NO_x_ directly contributes to NO₂ formation and PM₁₀ arises from similar emission processes. In this study, we formulate the task as daily NO₂ estimation (nowcasting), using contemporaneous meteorology and co-pollutant inputs ($\:{x}_{t}$) measured on the same day. Consequently, the reported results reflect same-day mapping performance rather than multi-step-ahead forecasting, which would require lagged predictors. Daily observations for all the variables were obtained from the Air Quality Monitoring Center operated by the Istanbul Metropolitan Municipality^47^. The input and output variables used in the model have a daily data frequency, covering the period from 1 January 2019 to 31 December 2023, corresponding to 1,826 calendar days.

**Fig. 1:**
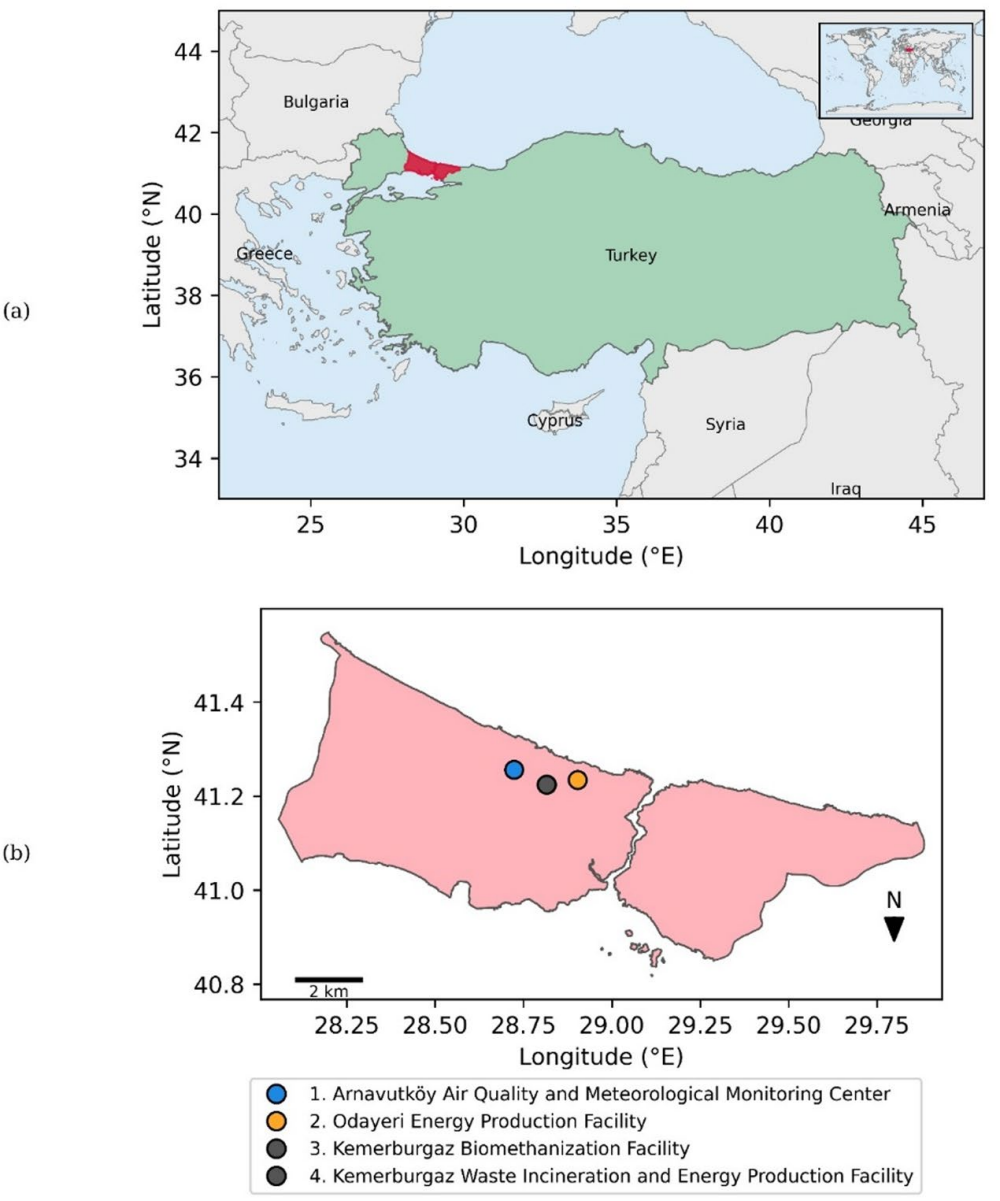
Distributions of biomass power plants and meteorological and air monitoring stations in the study area. The map was created by the authors using Python 3.14.2 (https://www.python.org/) with GeoPandas 1.1.3 (https://geopandas.org/) and Matplotlib 3.10.8 (https://matplotlib.org/). Base boundary data were obtained from Natural Earth, and additional geospatial boundary data were derived from the sources specified in the mapping script archived in the public code repository.

The spatial distributions of the biomass power plants and the meteorological and air quality monitoring stations are shown in Fig. 1.

The biomass power plants considered in this study are located within the Eyüp district, close to the administrative boundary with Arnavutköy. The nearest air quality and meteorological monitoring station is also located in Arnavutköy and daily observations from this station were used as model inputs.

Records with missing daily observations for any variable were removed. To mitigate the influence of sensor-related anomalies, extreme outliers were filtered using a data-driven rule based on the empirical distribution of each variable. Following these data-cleaning steps, all input and output variables were normalized to the [0, 1] range using min–max scaling. Min–max scaling parameters were fitted on the training subset only and then applied unchanged to the validation and test subsets. This normalization strategy prevents data leakage and ensures stable convergence during optimization.

## Data quality control and statistical test results

The standard normal homogeneity test (SNHT) and Mann–Kendall trend test were applied to each meteorological and air quality input variable (solar radiation, air temperature, relative humidity, NOₓ and PM₁₀) to assess data quality. These tests evaluate homogeneity (stationarity) and the presence of monotonic trends in the time series. The determination of any inhomogeneities or significant trends is important because such patterns can bias model training and prediction.

The dataset comprises daily observations from 1 January 2019 to 31 December 2023. The data were split chronologically into training (70% of records), validation (15%) and testing (15%) subsets. Because the data are time-ordered, k-fold cross-validation was not used; the chronological test set was kept untouched and used only once for the final comparison. This design ensures an unbiased evaluation under a realistic deployment setting.

The results of the homogeneity and trend analyses are summarized in Supplementary Table S1. None of the variables, i.e., solar radiation, air temperature, NO_x_, relative humidity and PM₁₀, followed normal distributions in any of the three subsets. According to the results of the Mann–Kendall test, no statistically significant monotonic trend was detected in the training data, whereas all the variables exhibited significant trends in the validation data. In the testing data, a trend was observed for solar radiation and PM₁₀, whereas air temperature, NO_x_ and relative humidity did not show any significant trends.

These findings reveal the following two key challenges for predictive modelling: the widespread nonnormality of input distributions and the variability of trend structures across temporal segments. Therefore, the prediction models must be robust to nonnormal distributions and able to handle changes in trend behaviour across the training, validation and testing periods.

## Methodology

The ANFIS, first introduced by Jang^48^, is a hybrid technique that integrates ANNs with fuzzy inference systems (FISs) to address challenges such as modelling, control and parameter estimation in complex systems^49^. Combining the strengths of ANNs and fuzzy set theory, it mitigates the main limitations inherent to each individual approach and allows membership function and rule parameters to be learned directly from data without complete reliance on expert-defined rule bases. In this way, it jointly processes numerical and linguistic information, preserving the classification and pattern recognition capabilities of ANNs while benefiting from the interpretability and transparency offered by fuzzy logic. Compared with conventional ANN models, the ANFIS is more user friendly, reduces the risk of memorization errors and, owing to its adaptability, ability to handle nonlinear problems and fast learning capability, provides a flexible and powerful tool for a wide range of applications^50^.

**Fig. 2:**
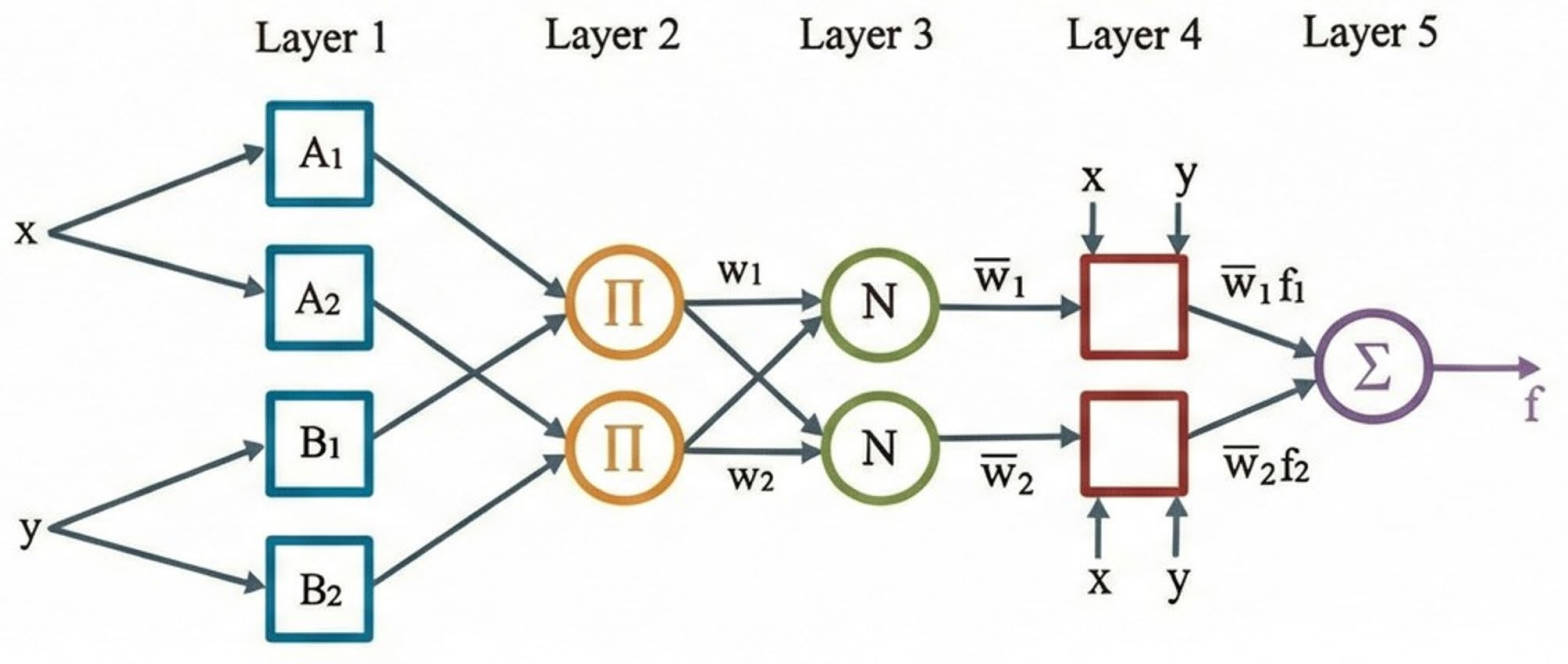
ANFIS architecture.

The ANFIS provides an FIS by extracting membership function (MF) parameters directly from the training data. Among FISs, Mamdani and Sugeno structures are the most commonly used^51^. Figure 2 illustrates the architecture of the ANFIS model, which comprises two inputs, one output and two rules^52^. In the first layer, each node corresponds to a specific membership function; the second layer determines the firing strength of each rule through multiplication; the third layer normalizes these firing strengths; the fourth layer computes each rule’s contribution to the overall output; and the fifth layer aggregates these contributions to generate the final output.

## Proposed model

The ANNHFIS-PSO model proposed in this study combines the predictive power of an ANN with a fuzzy inference mechanism based on NHFS. The model employs a Hamacher T-norm-based aggregation for the fusion of dual Gaussian membership functions and uses PSO to optimize both the antecedent membership-function parameters and the rule-weight coefficients. Finally, gradient-based fine-tuning with the Adam optimizer further improves the model’s performance. The mathematical framework and theoretical foundations of the model are described in detail in the following sections. Formally, the estimation problem represents the mapping of contemporaneous meteorological and pollutant variables to the target NO₂ concentration as given in Eq. (1).

1 $$\:\widehat{N{O}_{2}}\left(t\right)=f\left(SR\left(t\right),\:\:T\left(t\right),\:\:RH\left(t\right),\:\:N{O}_{x}\left(t\right),\:\:P{M}_{10}\left(t\right)\right)$$

Here, $\:\widehat{N{O}_{2}}\left(t\right)$ denotes the estimated daily mean nitrogen dioxide concentration ($\:{\upmu\:}\mathrm{g}/{\mathrm{m}}^{3}$). Among the inputs, $\:SR\left(t\right)$ represents the daily mean solar radiation $\:W/{m}^{2},\:\:T\left(t\right)$ is the air temperature $\:(^\circ\:C)$ and $\:RH\left(t\right)$ is the relative humidity (%). Additionally, $\:N{O}_{x}\left(t\right)$ and $\:P{M}_{10}\left(t\right)$ denote the concentrations of nitrogen oxides and particulate matter ($\:{\upmu\:}\mathrm{g}/{\mathrm{m}}^{3}$), respectively, measured contemporaneously at day t.

## Model structure and rule base

Each fuzzy rule is defined in a first-order Sugeno format that combines input membership conditions with a linear output expression, as expressed in Eq. (2):

2 $$\:{R}_{k}=If\:{x}_{1}\:is\:{A}_{1}^{k}\:and\:{x}_{2\:}is\:{A}_{2}^{k}\dots\:then\:{y}_{k}=\:\sum\:_{{j}_{1}}^{n}{a}_{kj}{x}_{j}+{r}_{k}$$

where.

- $\:{x}_{j}$ denotes the j-th input variable used in the antecedent part of the fuzzy rule;
- $\:{A}_{j}^{k}$ represents the fuzzy set associated with input $\:{x}_{j}$ in rule k; and.
- $\:{a}_{kj}$ is the consequent parameter (linear coefficient) for the input $\:{x}_{j}$ in rule k and $\:{r}_{k}$ is the bias term (offset) of the Sugeno output.

### Rule-base construction and number of rules

We construct the fuzzy rule base using a full Cartesian (grid) design. With $\:n=5$ inputs and three linguistic terms per input (Low, Medium, High), this yields $\:K={3}^{5}=243$ first-order Sugeno rules covering all antecedent label combinations. The NHFS fuzzification used to compute memberships (via the $\:T$, $\:I$ and $\:F$ components) is described next.

### Pruning/selection and effective sparsity

No explicit pruning or rule selection is applied; all $\:K=243$ rules are retained during training and inference. In practice, non-informative rules contribute negligibly due to normalized activations and the PSO-optimized rule weights $\:{\omega\:}_{k}$. Inference cost scales linearly with $\:K$; for $\:K=243$ and five inputs, computation remains tractable for daily-scale modelling.

### Fuzzification using neutrosophic hesitant fuzzy sets

Each input $\:x\:\in\:\:{R}^{n}$ is decomposed into the following three components: truth ($\:{\mu\:}_{T}$), indeterminacy ($\:{\mu\:}_{I}$) and falsity ($\:{\mu\:}_{F}$).

Unlike classical fuzzy ANFIS, which represents uncertainty with a single membership grade $\:\mu\:\left(x\right)$, NHFS models uncertainty via three explicitly separated components (T/I/F), parameterized as $\:\left({\mu\:}_{T}\left(x\right),{\mu\:}_{I}\left(x\right),{\mu\:}_{F}\left(x\right)\right)$. This differs from intuitionistic fuzzy formulations, where the hesitation/indeterminacy degree is not an independent component but is derived under the constraint $\:\mu\:\left(x\right)+\nu\:\left(x\right)\le\:1$, with $\:\pi\:\left(x\right)=1-\mu\:\left(x\right)-\nu\:\left(x\right)$. It also differs from hesitant fuzzy formulations, which represent hesitation as a set of possible membership grades for the membership function, without explicitly separating T and F into distinct components. In our design, hesitation is retained by using dual-Gaussian candidates for each neutrosophic component and fusing them via the Hamacher operator, yielding smooth and numerically stable membership functions. Under this construction, $\:{\mu\:}_{I}$captures ambiguous/uncertain evidence, while $\:{\mu\:}_{T}$ and $\:{\mu\:}_{F}$ allow supporting and contradicting evidence to be expressed explicitly.

For each component, two Gaussian membership functions (with parameters m and σ) are used to represent its fuzzy membership degree and are given by Eq. (3), where $\:m$ and $\:\sigma\:$ denote the mean and standard deviation of the Gaussian function, respectively.

3 $$\:\mu\:\left(x;m,\sigma\:\right)=exp\left(-\frac{{\left(x-m\right)}^{2}}{{2\sigma\:}^{2}}\right)$$

Two Gaussian membership functions are assigned to each neutrosophic component (T, I, and F) as lower/upper candidates to capture the hesitant nature of NHFS, thereby allowing two plausible membership degrees rather than a single crisp membership curve. The resulting pair is then fused using the Hamacher T-norm Eqs. (4)-(6) to ensure smooth transitions in overlapping regions. Because all inputs are min–max normalized to [0, 1], the Gaussian parameters are bounded during PSO to ensure numerical stability and a feasible search space (m ∈ [0.0, 1.1], σ ∈ [0.03, 0.5]). During the subsequent Adam-based fine-tuning stage, σ was additionally clipped to [1 × 10⁻³, 1.1] to prevent numerical instabilities. Each membership function in the ANNHFIS-PSO model has 12 parameters (two Gaussian functions for each of the three neutrosophic components). With five input variables (and three membership functions per input), this results in a total of 180 antecedent parameters, which were optimized during the PSO-based global search and subsequently fine-tuned using Adam.

Several alternative combination operators (geometric mean, algebraic product, nilpotent minimum, Dombi, Lukasiewicz, Einstein, Yager, maximum, average and weighted average) were also tested. The Hamacher T-norm produced the smoothest gradients and the best performance in regions with overlapping membership functions, which justifies its use in Eqs. (4)–(6).

4 $$\:{\mu\:}_{T}\left(x\right)=\frac{{\mu\:}_{{T}_{1}}\left(x\right).{\mu\:}_{{T}_{2}}\left(x\right)}{{\mu\:}_{{T}_{1}}\left(x\right)+{\mu\:}_{{T}_{2}}\left(x\right)-{\mu\:}_{{T}_{1}}\left(x\right).{\mu\:}_{{T}_{2}}\left(x\right)+\epsilon\:}$$

5 $$\:{\mu\:}_{I}\left(x\right)=\frac{{\mu\:}_{{I}_{1}}\left(x\right).{\mu\:}_{{I}_{2}}\left(x\right)}{{\mu\:}_{{I}_{1}}\left(x\right)+{\mu\:}_{{I}_{2}}\left(x\right)-{\mu\:}_{{I}_{1}}\left(x\right).{\mu\:}_{{I}_{2}}\left(x\right)+\epsilon\:}$$

6 $$\:{\mu\:}_{F}\left(x\right)=\frac{{\mu\:}_{{F}_{1}}\left(x\right).{\mu\:}_{{F}_{2}}\left(x\right)}{{\mu\:}_{{F}_{1}}\left(x\right)+{\mu\:}_{{F}_{2}}\left(x\right)-{\mu\:}_{{F}_{1}}\left(x\right).{\mu\:}_{{F}_{2}}\left(x\right)+\epsilon\:}$$

where ε is a small constant that prevents division by zero. Finally, a raw NHFS score is obtained by aggregating the three component values $\:T$, $\:I$ and $\:F$, as shown in Eq. (7).

7 $$\:{NHF}_{raw}\left(x\right)={\mu\:}_{T}\left(x\right)+{\mu\:}_{I}\left(x\right)-{\mu\:}_{F}\left(x\right)$$

Although the membership degrees satisfy $\:{\mu\:}_{T}\left(x\right),{\:\mu\:}_{I}\left(x\right),\:{\mu\:}_{F}\left(x\right)\in\:[0,\:1]$, the raw score computed in Eq. (7) can theoretically fall outside the unit interval (i.e., within $\:\left[-1,\:\:2\right]$). Therefore, before rule activation, we clip the score to $\:[0,\:1]$ as defined in Eq. (8).

8 $$\:NHF\left(x\right)=min(1,\:max(0,\:{NHF}_{raw}\left(x\right)\left)\right)$$

The resulting clipped score $\:NHF\left(x\right)$ is subsequently used to compute the rule firing strengths.

### Rule activation, weighting and normalization

Each rule’s firing strength is computed as the product of the NHFS scores of all the input conditions Eq. (9):

9 $$\:{w}_{k}=\prod\:_{j=1}^{n}{NHF}_{kj}\left({x}_{j}\right)$$

We assign each rule a weight $\:{\omega\:}_{k}$ (optimized via PSO) and the weighted activation is given by Eq. (10):

10 $$\:{\stackrel{\sim}{w}}_{k}={w}_{k}.{\omega\:}_{k}$$

#### Note

PSO optimizes the rule weights $\:{\omega\:}_{k}$ to realize soft relevance weighting (without hard pruning), keeping the rule count fixed ($\:K=243$) while attenuating the contribution of less relevant rules in the final output.

The rule activation scores are then normalized to ensure that their total contribution across all rules sums to one, providing proportional weights for the output computation, as expressed in Eq. (11):

11 $$\:{\stackrel{-}{w}}_{k}=\frac{{\stackrel{\sim}{w}}_{k}}{{\sum\:}_{k=1}^{R}{\stackrel{\sim}{w}}_{k}+\epsilon\:}$$

where $\:\epsilon\:$ is a small positive constant added to avoid division by zero.

### Sugeno-type consequent evaluation and output aggregation

Each rule i yields a linear output modelled as a function of the input variables, as described in Eq. (12):

12 $$\:{y}_{k}=\sum\:_{j=1}^{n}{a}_{kj}{x}_{j}+{r}_{k}$$

The weighted contribution of each rule to the system output is given in Eq. (13):

13 $$\:{\stackrel{-}{y}}_{k}={\stackrel{-}{w}}_{k}.{y}_{k}$$

The final output of the system is computed by summing the weighted outputs of all the rules, as expressed in Eq. (14):

14 $$\:y=\sum\:_{k=1}^{R}{\stackrel{-}{y}}_{k}$$

To ensure numerical stability and comply with application-specific constraints, the final output is constrained to the range [0, 1] (Eq. (15)) so that it remains within the normalized bounds.

15 $$\:{y}_{final}=clip\left(y,\mathrm{0,1}\right)$$

### PSO-based optimization of MF parameters and rule weights

In the proposed ANNHFIS-PSO model, the optimization of both the Gaussian membership function parameters—mean (m) and standard deviation ($\:\sigma\:$)—and the rule weights ($\:{\omega\:}_{k}$) was performed using the PSO algorithm. Each particle in the PSO population represents a potential solution that encodes all the MF parameters and corresponding rule weights.

The optimization process aimed to enhance the generalization performance of the model by maximizing the validation R² score. To prevent overfitting and improve generalization during the global optimization process, a penalized objective function was employed, as expressed in Eq. (16). This function balances model accuracy between the training and validation datasets by introducing a penalty term that discourages overfitting. In this equation, $\:{R}_{Train}^{2}$ and $\:{R}_{Val}^{2}$ represent the R² scores for the training and validation sets, respectively and $\:\lambda\:$ is a user-defined penalty coefficient that controls the trade-off between these two objectives.

16 $$\:Objective={R}_{Val}^{2}-\lambda\:.\mathrm{m}\mathrm{a}\mathrm{x}(0,{R}_{Train}^{2}-{R}_{Val}^{2})$$

**Fig. 3:**
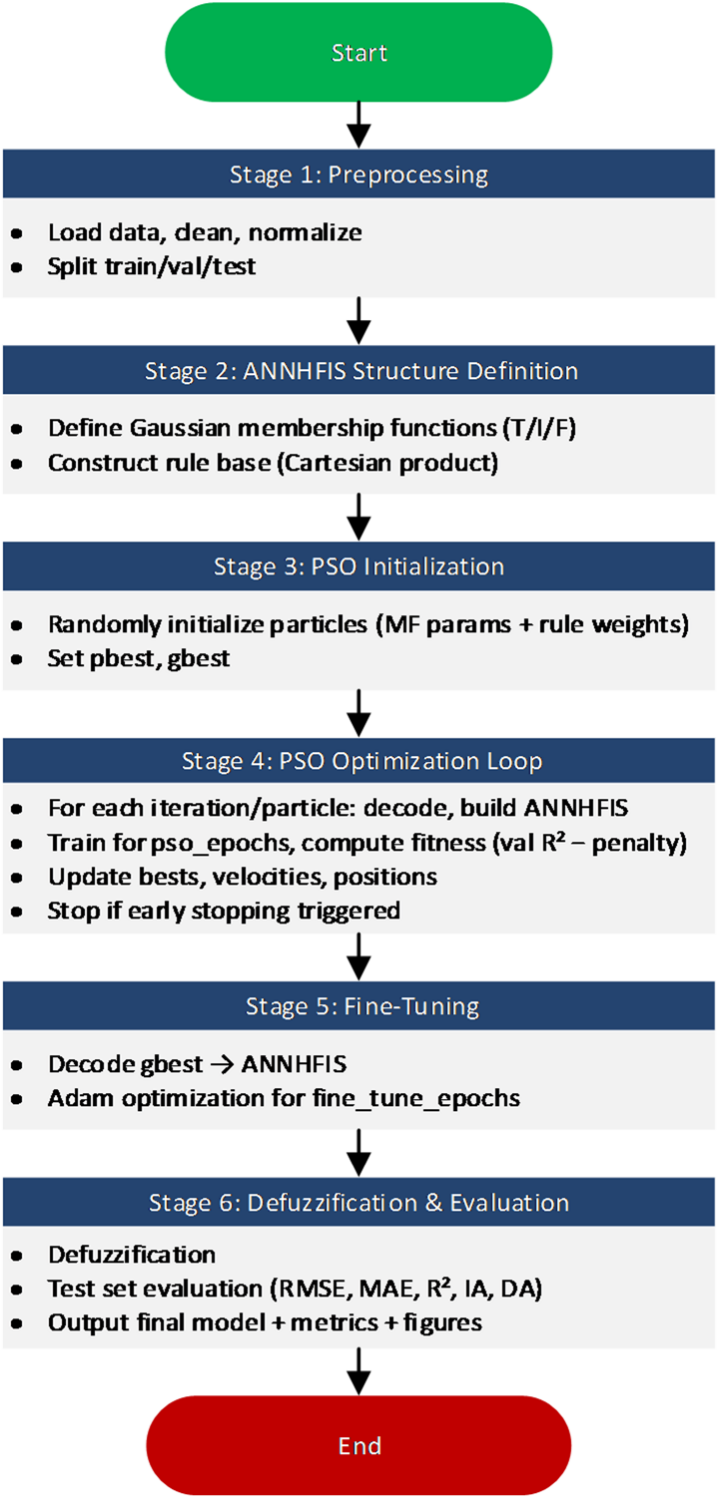
General workflow of the proposed ANNHFIS-PSO model.

The overall training and optimization workflow is summarized in Fig. 3 and the complete end-to-end procedure is provided in Algorithm 1.

Following global optimization, a fine-tuning step was performed using the Adam optimizer to refine the MF parameters. This gradient-based local search adjusts the m and σ values on the basis of the backpropagation of output errors and improves the model’s accuracy.

A regularization term was also applied during the fine-tuning stage to prevent overfitting. Moreover, all rule weights $\:{\omega\:}_{k}$ were constrained within the range [0, 1] and the MF parameters were bounded to ensure numerical stability. The PSO search bounds for the Gaussian parameters are $\:m\in\:[0.0,\:1.1]$. For $\:\sigma\:$, the PSO search used $\:\sigma\:\in\:[0.03,\:0.5]$ for each of the two Gaussians per neutrosophic component. During the subsequent Adam-based fine-tuning stage, $\:\sigma\:$ was additionally clipped to $\:\left[1\times\:{10}^{-3},1.1\right]$ for numerical stability.

### Representative rules

The proposed ANNHFIS-PSO model employs a full Cartesian first-order Sugeno rule base with three linguistic terms per input and five inputs, yielding $\:K={3}^{5}=243$ rules. While the rule structure is fixed, the NHFS-based membership parameters and the rule weights are optimized during training, and the consequent coefficients $\:\left\{{a}_{k,j},{r}_{k}\right\}$ are learned from data.

To illustrate interpretability, we present two representative rules corresponding to contrasting air-quality conditions. As an illustrative high-pollution example, Rule $\:k$ is defined as:

IF Solar Radiation is Low AND Air Temperature is Low AND Relative Humidity is High AND $\:{\mathrm{N}\mathrm{O}}_{x}$ is High AND $\:{\mathrm{P}\mathrm{M}}_{10}$ is High, THEN

$$\:{y}_{k}={r}_{k}+{a}_{k,1}\left(\mathrm{Solar}\right)+{a}_{k,2}\left(\mathrm{Temp}\right)+{a}_{k,3}\left(\mathrm{Humidity}\right)+{a}_{k,4}\left({\mathrm{NO}}_{x}\right)+{a}_{k,5}\left({\mathrm{PM}}_{10}\right)$$

This antecedent describes conditions commonly associated with poor air quality (reduced dispersion under low radiation/temperature combined with elevated precursor levels). Conversely, as a cleaner-air example, Rule $\:m$ is defined as:

IF Solar Radiation is High AND Air Temperature is Medium AND Relative Humidity is Low AND $\:{\mathrm{N}\mathrm{O}}_{x}$ is Low AND $\:{\mathrm{P}\mathrm{M}}_{10}$ is Low, THEN

$$\:{y}_{m}={r}_{m}+{a}_{m,1}\left(\mathrm{Solar}\right)+{a}_{m,2}\left(\mathrm{Temp}\right)+{a}_{m,3}\left(\mathrm{Humidity}\right)+{a}_{m,4}\left({\mathrm{NO}}_{x}\right)+{a}_{m,5}\left({\mathrm{PM}}_{10}\right)$$

The linguistic terms (Low, Medium, High) correspond to NHFS-based Gaussian membership functions, where the $\:T$, $\:I$, and $\:F$ memberships are formed by combining two Gaussian functions via the Hamacher operator. A single NHFS score is then computed and clipped to $\:[0,\:1]$ (Eq. (8)) to ensure numerical stability before rule aggregation.

During inference, each rule’s firing strength is computed, weighted, and normalized; the final prediction is obtained by aggregating the rule consequents, where each rule contributes proportionally to its normalized firing strength Eqs. (9)–(14).

To further clarify the stability and practical interpretability of the learned membership functions and rules, several mechanisms are used to ensure numerical stability and a feasible search space. Specifically, all inputs/outputs are min–max normalized to [0, 1]; the Gaussian MF parameters are bounded during PSO $\:\left(m\in\:[0.0,\:1.1],{\hspace{0.17em}}\sigma\:\in\:[0.03,\:0.5]\right)$ and, during the subsequent Adam-based fine-tuning stage, $\:\sigma\:$ was additionally clipped to $\:\left[1\times\:{10}^{-3},{\hspace{0.17em}}1.1\right]$to prevent numerical instabilities. In practice, no explicit pruning or rule selection is applied and the full Cartesian rule base is retained (K = 243); however, PSO optimizes the rule weights to realize soft relevance weighting (without hard pruning). Consequently, non-informative rules contribute negligibly due to normalized firing strengths, thereby preserving the transparent first-order Sugeno structure with Low/Medium/High antecedents.

### Implementation details and experimental setup

All experiments were executed in a unified Google Colab Pro+ (Intel Xeon central processing unit (CPU), NVIDIA A100 graphics processing unit (GPU)) environment. The models were implemented in Python 3 using NumPy, pandas, scikit-learn, Ray, CuPy, Matplotlib, and Seaborn. The specific hyperparameters and training procedures defined in Algorithm 1 were utilized for the proposed model. To ensure numerical stability, output values were clipped to the $\:[0,\:1]\:\:$range during training. Early stopping was employed to prevent overfitting: patience = 5 was set for particle evaluation inside the PSO loop and patience = 3 was used during the subsequent Adam-based fine-tuning stage, with a minimum delta of $\:1\times\:{10}^{-6}$ for both. Additionally, the PSO outer loop utilized a specific early-stopping rule (PSO_PATIENCE = 5, PSO_MIN_DELTA = $\:1\times\:{10}^{-6}$) to optimize the search budget. The complete hyperparameter configuration of the proposed method and all benchmark models, together with the end-to-end wall-clock runtime, is reported in Supplementary Table S2.


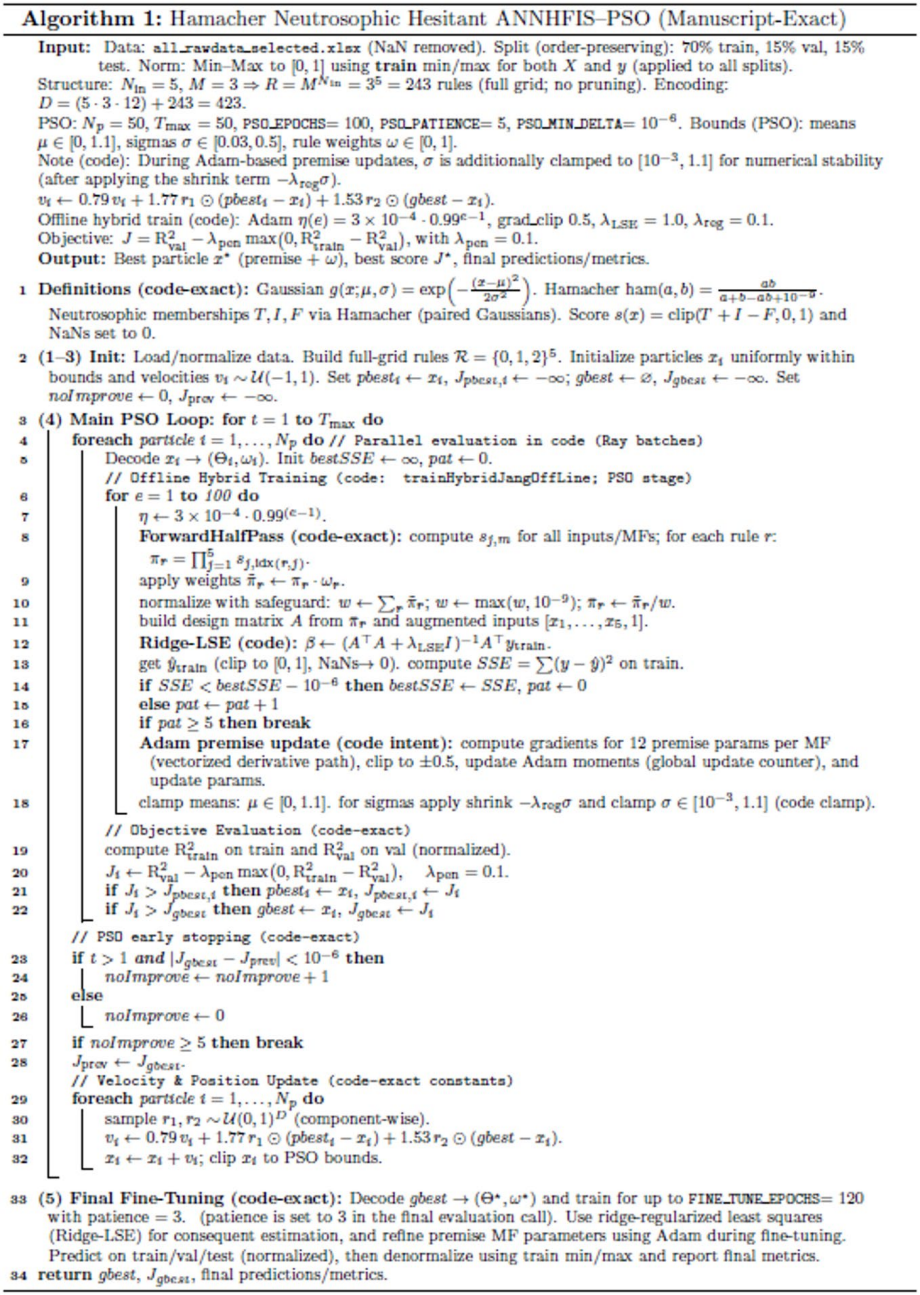


### Evaluation metrics

Model performance was evaluated on each subset using root mean square error (RMSE), mean absolute error (MAE), coefficient of determination (R²), index of agreement (IA), and degree of agreement (DA). These metrics are defined in Eqs. (17)–(21).

17 $$\:RMSE=\sqrt{\frac{1}{n}\sum\:_{i=1}^{n}{\left({y}_{i}-{\widehat{y}}_{i}\right)}^{2}}$$

18 $$\:MAE=\frac{1}{n}\sum\:_{i=1}^{n}\left|{y}_{i}-{\widehat{y}}_{i}\right|$$

19 $$\:{R}^{2}=1-\frac{\sum\:_{i=1}^{n}{\left({y}_{i}-{\widehat{y}}_{i}\right)}^{2}}{\sum\:_{i=1}^{n}{\left({y}_{i}-\stackrel{-}{y}\right)}^{2}}$$

20 $$\:IA=1-\frac{\sum\:_{i=1}^{n}{\left({y}_{i}-{\widehat{y}}_{i}\right)}^{2}}{\sum\:_{i=1}^{n}{\left(\left|{\widehat{y}}_{i}-\stackrel{-}{y}\right|+\left|{y}_{i}-\stackrel{-}{y}\right|\right)}^{2}}$$

21 $$\:DA=1-\frac{\sum\:_{i=1}^{n}\left|{y}_{i}-{\widehat{y}}_{i}\right|}{\sum\:_{i=1}^{n}{\left(\left|{\widehat{y}}_{i}-\stackrel{-}{y}\right|+\left|{y}_{i}-\stackrel{-}{y}\right|\right)}^{2}}$$

### Benchmark models

To quantify the improvement delivered by the proposed ANNHFIS-PSO model, we implemented five benchmarks: grid-search-tuned ANNHFIS (ANNHFIS-GS), ANFIS-PSO, ANFIS-GS, multilayer perceptron artificial neural network (MLP-ANN), and LSTM. All benchmarks used the same input variables and predicted the same target (NO₂) as the proposed model. Hyperparameters for each method were tuned on the same chronological train/validation split and the configuration achieving the highest validation $\:{R}^{2}$ was retained. The complete search spaces and the selected final settings for all methods are summarized in Supplementary Table S2.

To mitigate optimizer-related bias, we evaluate ANFIS and ANNHFIS under both GS and PSO, enabling within-family comparisons in which the optimizer is held fixed. For MLP-ANN and LSTM, GS is applied over commonly adopted architectural and training hyperparameters.

### ANNHFIS-GS

The ANNHFIS-GS baseline was based on a first-order Sugeno ANNHFIS in which each input was represented by three neutrosophic components (T/I/F) and each component was parameterized by two Gaussian membership functions (i.e., six Gaussians per input). A grid search (GS) was applied over both the MF initialization settings and key learning hyperparameters. MF initialization was varied by shifting each feature’s normalized mean using $\:{\Delta\:}\in\:\{0.05,\:0.10,\:0.15\}$ and scaling its standard deviation using $\:{\sigma\:}_{\mathrm{factor}}\in\:\{0.8,\:0.9,\:1.0,\:1.1,\:1.2\}$. The learning rate and regularization terms were also tuned over discrete candidate sets. Each candidate was trained using Jang’s hybrid learning for up to 500 epochs with early stopping (patience = 10).

### ANFIS-PSO

We employed a first-order Sugeno ANFIS with two Gaussian membership functions per input variable. The Gaussian antecedent parameters (mean $\:m$ and standard deviation $\:\sigma\:$) were optimized using particle swarm optimization (PSO) with 50 particles over 50 iterations, within $\:m\in\:[0,\:1.1]$ and $\:\sigma\:\in\:[0.03,\:0.5]$. During PSO, each candidate solution was evaluated using a nested hybrid learning procedure (up to 150 epochs) to compute the fitness reliably. After PSO convergence, the global-best solution was further refined via an Adam fine-tuning stage (50 epochs with early stopping, patience = 3).

### ANFIS-GS

We employed a first-order Sugeno ANFIS with two Gaussian membership functions per input variable. The Gaussian antecedent parameters were optimized using an exhaustive grid search (GS) over the MF placement settings, with $\:{\Delta\:}\in\:\{0.05,\:0.10,\:0.15\}\:$and $\:{\sigma\:}_{\mathrm{f}\mathrm{a}\mathrm{c}\mathrm{t}\mathrm{o}\mathrm{r}}\in\:\{0.8,\:0.9,\:1.0,\:1.1,\:1.2\}$. During GS, each candidate configuration was trained using Jang’s hybrid learning procedure (least squares estimation for consequents and backpropagation for antecedents) for up to 100 epochs with early stopping (patience = 10, min_delta = $\:1\times\:{10}^{-6}$), using a learning rate of 0.01 and $\:{\lambda\:}_{\mathrm{r}\mathrm{e}\mathrm{g}}=1\times\:{10}^{-4}$.

### MLP-ANN

We employed Scikit-Learn’s MLPRegressor to construct a feedforward neural network with two hidden layers. The model hyperparameters were optimized using an exhaustive grid search (GS) over hidden-layer sizes $\:\left\{(100,\:50),(200,\:100)\right\}$, activation functions $\:\left\{\mathrm{R}\mathrm{e}\mathrm{c}\mathrm{t}\mathrm{i}\mathrm{f}\mathrm{i}\mathrm{e}\mathrm{d}\:\mathrm{L}\mathrm{i}\mathrm{n}\mathrm{e}\mathrm{a}\mathrm{r}\:\mathrm{U}\mathrm{n}\mathrm{i}\mathrm{t}\:\left(\mathrm{R}\mathrm{e}\mathrm{L}\mathrm{U}\right),\mathrm{t}\mathrm{a}\mathrm{n}\mathrm{h}\right\}$ and solvers {Adam, Stochastic Gradient Descent (SGD)}. Each candidate configuration was trained for up to 1000 epochs using a fixed random seed for reproducibility.

### LSTM

We built an LSTM network in Keras with one or two recurrent layers followed by a dense output neuron. The input data were reshaped to (samples, time steps = 1, features = 5), i.e., a single time step consistent with same-day estimation (nowcasting) rather than multi-step-ahead forecasting. A GS was conducted over the number of units [25, 32, 40, 50, 64], number of layers [1, 2], batch size [8, 16, 32, 64] and epochs [50, 60, 100, 150, 200], using the Adam optimizer and mean squared error (MSE) loss.

## Results

### Comparison analysis

**Fig. 4:**
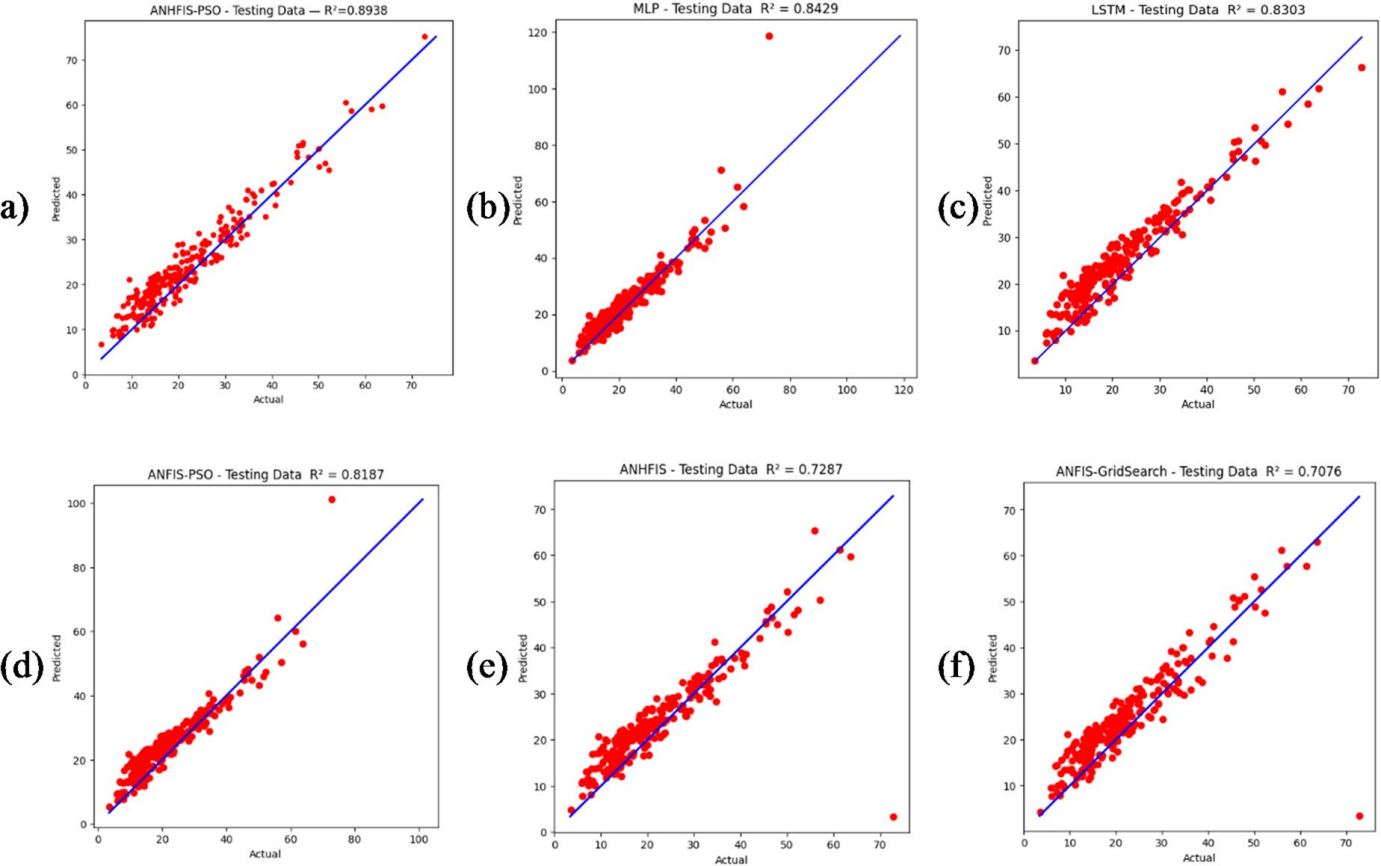
Scatter plots of observed versus predicted daily NO₂ concentrations on the test dataset for each model: (**a**) ANNHFIS-PSO, (**b**) MLP-ANN, (**c**) LSTM, (**d**) ANFIS-PSO, (**e**) ANNHFIS-GS and (**f**) ANFIS-GS.

**Fig. 5:**
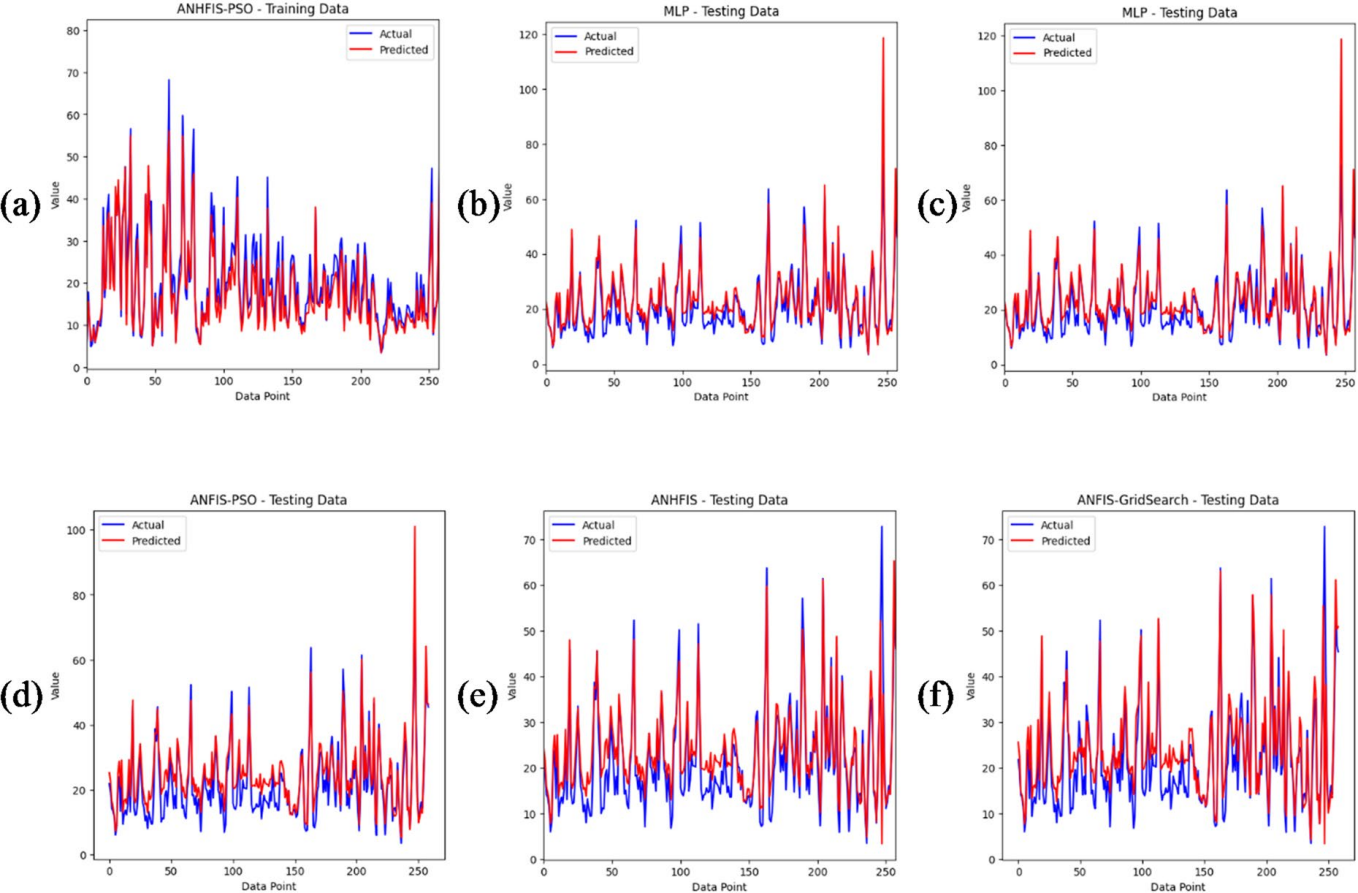
Time series comparison of observed and predicted daily NO₂ concentrations on the test dataset: (**a**) ANNHFIS-PSO, (**b**) MLP-ANN, (**c**) LSTM, (**d**) ANFIS-PSO, (**e**) ANNHFIS-GS and (**f**) ANFIS-GS.

**Fig. 6:**
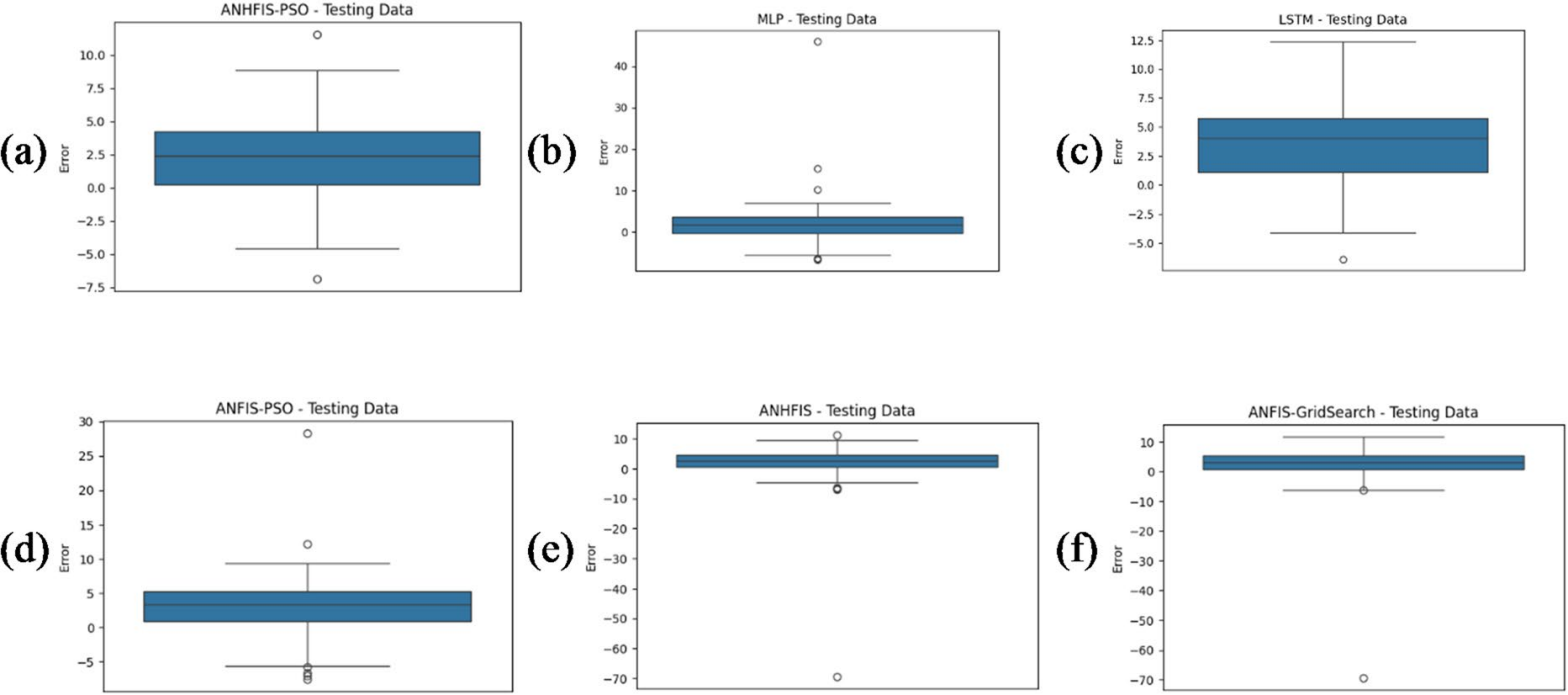
Box plots of residuals (observed minus predicted NO₂) on the test dataset for each model: (**a**) ANNHFIS-PSO, (**b**) MLP-ANN, (**c**) LSTM, (**d**) ANFIS-PSO, (**e**) ANNHFIS-GS and (**f**) ANFIS-GS.

In this study, the predictive performance of the proposed ANNHFIS-PSO model was compared with that of benchmark approaches, including MLP-ANN, LSTM, ANFIS-PSO, ANFIS-GS and ANNHFIS-GS. All models were trained and evaluated on the same daily NO₂ dataset and their performance was quantified using multiple statistical metrics, namely RMSE, R², MAE, IA and DA. Parameter optimization was carried out using either PSO or GS. The numerical results are summarized in Table 1, while Figs. 4, 5 and 6 present the corresponding time-series and error plots.

**Table 1:** Summary of performance metrics on the test dataset.

Method	Search/optimizer	RMSE (µg/m³)	*R*²	MAE (µg/m³)	IA	DA
ANNHFIS	**PSO**	3.6488	0.8938	3.0460	0.9718	0.8179
MLP-ANN	**GS**	4.4387	0.8429	2.9563	0.9613	0.8233
LSTM	**GS**	4.6128	0.8303	3.9246	0.9550	0.7654
ANFIS	**PSO**	4.7690	0.8187	3.8595	0.9517	0.7693
ANNHFIS	**GS**	5.8330	0.7287	3.5593	0.9191	0.7873
ANFIS	**GS**	6.0555	0.7076	3.8449	0.9189	0.7702

To assess whether the observed differences are statistically significant, we conducted a paired Wilcoxon signed-rank test on the per-day absolute errors on the test set (α = 0.05; one-sided alternative: ANNHFIS-PSO yields smaller errors). The proposed model showed statistically significant improvements over LSTM, ANFIS-PSO, ANFIS-GS and ANNHFIS-GS (*p* < 0.05), whereas no statistically significant advantage in absolute errors was observed against MLP-ANN (*p* > 0.05), consistent with their close MAE values.

On the test set, ANNHFIS-PSO achieved the lowest RMSE = 3.6488 µg/m³ and the highest R² = 0.8938 and IA = 0.9718 among the evaluated models, as reported in Table 1, while its MAE = 3.0460 µg/m³ and DA = 0.8179 were comparable to those of the benchmark models. As shown in Figs. 4(a)-6(a), the predictions are generally aligned with the 1:1 line, capture short-term fluctuations and peak NO₂ concentrations to a reasonable extent and show a relatively narrow residual distribution with limited outliers, suggesting consistency in capturing the nonlinear dynamics of NO₂ under the present conditions. This robustness is consistent with NHFS’s explicit modeling of indeterminacy and conflicting evidence through separated T/I/F components. Accordingly, under identical PSO optimization conditions, ANNHFIS-PSO improves over ANFIS-PSO by reducing RMSE from 4.7690 to 3.6488 µg/m³ and improving R² from 0.8187 to 0.8938. These gains are summarized in Table 1 and are further supported by the narrower residual distribution with fewer pronounced outliers in Fig. 6, whereas benchmark models exhibit wider residual spreads and outlier excursions.

The MLP-ANN model ranked second in overall performance. Compared with the ANNHFIS-PSO model, it produced higher RMSE = 4.4387 µg/m³ and lower R² = 0.8429 and IA = 0.9613 values, whereas its MAE = 2.9563 µg/m³ and DA = 0.8233 metrics were better than those of the ANNHFIS-PSO model, as reported in Table 1. An examination of Figs. 4(b)-6(b) reveals that its predictions deviate slightly more from the 1:1 reference line, particularly at higher NO₂ concentrations and exhibit visible yet moderate additional discrepancies during periods with large observed values.

Although the paired Wilcoxon signed-rank test indicated no statistically significant difference in per-day absolute errors between ANNHFIS-PSO and MLP-ANN (*p* > 0.05), the added model complexity is motivated by improved control of large-error events and structural transparency. First, ANNHFIS-PSO yields a lower RMSE and a higher R² than MLP-ANN, as reported in Table 1, which is particularly relevant in air-quality applications where underestimation during high-NO₂ episodes can be more consequential than small routine deviations. Second, the additional training overhead associated with PSO-based membership optimization is documented in Supplementary Table S2 and is accompanied by interpretability: unlike the black-box nature of MLP-ANN, ANNHFIS-PSO retains a transparent first-order Sugeno rule base with NHFS-based membership functions in the antecedents, enabling the extraction of human-readable linguistic rules, as illustrated in the Representative rules section. Accordingly, while MLP-ANN remains a lightweight option for resource-constrained deployments, ANNHFIS-PSO is positioned as a risk-aware and explainable alternative when peak-error control and model transparency are priorities.

The LSTM model achieved an intermediate performance among the tested methods. On the test set, it yielded RMSE = 4.6128 µg/m³, R² = 0.8303, MAE = 3.9246 µg/m³, IA = 0.9550 and DA = 0.7654, as reported in Table 1, indicating reasonably strong agreement with the observations but higher errors than those of ANNHFIS-PSO and MLP-ANN. An examination of Figs. 4(c)-6(c) reveals that its predictions remain broadly aligned with the 1:1 reference line and reproduce the overall temporal variability; however, deviations increase at higher NO₂ concentrations and during periods with pronounced peaks and the residuals exhibit a wider spread with several moderate outliers. Taken together, these results suggest that although the LSTM architecture is able to exploit temporal dependencies, its generalization capacity is limited for this relatively short daily NO₂ series, possibly due to the combination of a restricted sample size and the large number of trainable parameters.

Despite employing PSO-based optimization, the ANFIS-PSO model exhibited only moderate performance, with R² = 0.8187 and IA = 0.9517 and higher RMSE = 4.7690 µg/m³ and MAE = 3.8595 µg/m³ values than ANNHFIS-PSO, as reported in Table 1. As shown in Figs. 4(d)-6(d), the model generally reproduces the overall temporal patterns but underestimates several peaks, exhibits a broader scatter around the 1:1 line and yields residuals with a wider spread than those of the ANNHFIS-PSO model. These findings suggest that, in this study, an optimization scheme that focuses primarily on the consequent parameters within a conventional ANFIS structure may limit model flexibility and is associated with a more restricted predictive performance than the ANNHFIS-PSO architecture, which jointly adapts both antecedent and consequent parameters.

The ANNHFIS-GS model achieved lower accuracy, with R² = 0.7287 and IA = 0.9191 and had larger error statistics on the test set (RMSE = 5.8330 µg/m³; MAE = 3.5593 µg/m³), as reported in Table 1. As shown in Figs. 4(e)–6(e), the model exhibits more noticeable deviations from the 1:1 line and has difficulty reproducing extreme NO₂ levels, while the residual box plot shows a generally concentrated central distribution together with a pronounced negative outlier. Taken together, these findings suggest that, under the present configuration, the grid-search-based hyperparameter tuning adopted for ANNHFIS-GS, which explores the parameter space relatively coarsely, may be less flexible than the PSO-based optimization in ANNHFIS-PSO for handling the nonlinear and highly variable nature of the NO₂ time series in this study.

Among the benchmarks, the ANFIS-GS model produced the weakest results, with the highest RMSE = 6.0555 µg/m³ and the lowest R² = 0.7076 among all the methods, as reported in Table 1. As shown in Figs. 4(f)–6(f), its predictions exhibit wider scatter around the 1:1 line, a less consistent alignment with the observed time series and a broader residual distribution with several extreme values. Taken together, these findings indicate that, for this dataset and model configuration, the conventional ANFIS structure combined with GS offers limited robustness and predictive reliability compared with the other approaches.

Finally, to clarify whether the reported improvements stem from the proposed model structure or from the choice of optimizer, Table 1 provides optimizer-controlled comparisons within the same neuro-fuzzy family. When PSO is used for both models, ANNHFIS-PSO (RMSE = 3.6488, R² = 0.8938) outperforms ANFIS-PSO (RMSE = 4.7690, R² = 0.8187). A consistent advantage is also observed under GS, where ANNHFIS-GS (RMSE = 5.8330, R² = 0.7287) improves upon ANFIS-GS (RMSE = 6.0555, R² = 0.7076). Therefore, while the optimizer choice affects absolute accuracy, the performance advantage of ANNHFIS over ANFIS persists under the same optimization strategy, indicating that the gains are not solely attributable to PSO.

In summary, ANNHFIS-PSO achieved the lowest RMSE (3.6488 µg/m³) and the highest R² (0.8938) and IA (0.9718) on the test dataset, as reported in Table 1. Wilcoxon signed-rank tests on per-day absolute errors indicate significantly lower errors than LSTM, ANFIS-PSO, ANFIS-GS and ANNHFIS-GS (*p* < 0.05), whereas the difference versus MLP-ANN was not statistically significant (*p* > 0.05).

## Discussion

In air pollution prediction and modelling studies, model performance does not depend solely on the algorithm used. The choice of input variables, data quality and temporal resolution also play important roles, making direct comparisons of performance metrics across different studies difficult^53^.

In the current same-day NO₂ estimation setting, ANNHFIS-PSO achieved an RMSE of 3.6488 µg/m³, an MAE of 3.0460 µg/m³ and an R² of 0.8938, demonstrating favourable predictive performance. Relative to selected NO₂ studies reporting ANFIS^17^, PSO - slime mould algorithm - ANFIS (PSOSMA-ANFIS)^38^, bidirectional gated recurrent unit (Bi-GRU)^54^, principal component analysis - radial basis function network (PCA-RBF)^16^ and random forest (RF)^55^, ANNHFIS-PSO shows competitive error and R² levels.

On the other hand, some models, such as the MLP^56^ and improved complete ensemble empirical mode decomposition with adaptive noise - imperialist competitive algorithm - extreme learning machine (ICEEMDAN-ICA-ELM)^57^, report higher R² values (up to 0.96). These results suggest that different model architectures may be more suitable under different data and application conditions. However, beyond its predictive performance, the proposed ANNHFIS-PSO architecture illustrates how integrating NHFS into an ANFIS framework can enhance uncertainty modeling in same-day NO₂ estimation near biomass power plants. To the best of our knowledge, this represents the first ANFIS-based NHFS application reported for this problem.

Regarding computational efficiency, Supplementary Table S2 illustrates a trade-off between predictive performance and computational runtime. While the proposed ANNHFIS-PSO model entails a longer end-to-end runtime compared with lightweight baselines such as MLP-ANN and ANFIS-PSO, it remains notably more efficient than the LSTM benchmark. This increased computational demand is an important consideration for resource-constrained or time-sensitive monitoring deployments.

Taken together, these findings indicate that this study does not claim absolute superiority; instead, they position ANNHFIS-PSO as a competitive option for same-day NO₂ estimation in the vicinity of biomass power plants. Several limitations should be noted, which also guide directions for future work.

First, search budgets and optimization dynamics can differ across tuning strategies (e.g., PSO iterations versus GS combinations), which may influence absolute performance levels across models. Accordingly, future comparisons could adopt unified, budget-matched hyperparameter optimization (e.g., Bayesian optimization or equalized evaluation budgets) to strengthen cross-model fairness.

Second, scalability may become challenging as the number of inputs or linguistic terms increases. Retaining the full Cartesian rule base (K = 243) without explicit pruning is feasible in the present setting; however, the rule count grows exponentially with model dimensionality. This motivates exploring rule-reduction approaches (e.g., subtractive clustering / fuzzy C-means for data-driven rule generation, or post-training pruning of persistently low-activation rules) to maintain scalability while preserving accuracy.

Third, the empirical evaluation is intentionally designed as a focused case study (daily data from a single station in Istanbul and a single target pollutant, NO₂). While this controlled scope supports a rigorous assessment under well-defined conditions, it limits external validity. Accordingly, the reported performance should be interpreted as case-study evidence under the present setting rather than a guarantee of broad applicability. Further validation is warranted across multi-station networks and diverse regions, with extensions to additional pollutants (e.g., PM₂.₅, O₃, SO₂) and spatiotemporal modelling strategies.

Finally, this study focuses on same-day NO₂ estimation (nowcasting) using contemporaneous inputs. Extending the framework to forecasting (e.g., $\:t+1$) would require lagged predictors and forecasts of exogenous variables, implemented via either a recursive strategy (iterating one-step-ahead predictions) or a direct strategy (training horizon-specific models). Such extensions warrant further investigation.

### Conclusion and future work

Air pollution remains a critical challenge for public health and environmental sustainability. NO₂, in particular, requires close monitoring due to its adverse effects on respiratory health. Therefore, reliable estimation of NO₂ levels is critical for effective air quality management and protective planning.

The findings of this study suggest that the proposed ANNHFIS-PSO framework provides an effective approach for estimating daily NO₂ concentrations near biomass power plants in Istanbul. By integrating NHFS into the ANFIS architecture, the framework is designed to better handle data uncertainty than conventional methods. The observed error metrics and agreement indices across the evaluated benchmarks indicate that ANNHFIS-PSO may support decision-making for same-day NO₂ estimation in similar urban settings, where environmental data are complex and nonlinear.

Further validation is warranted across multi-station networks and diverse regions. An additional direction is to extend the framework toward multi-horizon forecasting by incorporating lagged predictors and exogenous forecasts.

## Supplementary Information

Below is the link to the electronic supplementary material.

Supplementary Material 1

Supplementary Material 2

Supplementary Material 3

## Data Availability

The raw air-quality observations used in this study are available online from the Istanbul Metropolitan Municipality Air Quality Monitoring Center at: https://havakalitesi.ibb.gov.tr/Pages/AirQuality. The curated dataset used for the analyses reported in this study is publicly available in Zenodo at 10.5281/zenodo.19024502.
