# Supplementary material for: A novel nowcasting (estimation) model based on an adaptive network neutrosophic hesitant fuzzy inference system (ANNHFIS): a case study of Istanbul
Source: Sci Rep. 2026 Mar 24;16:14855. doi: 10.1038/s41598-026-45618-7 (PMC13168669; doi:10.1038/s41598-026-45618-7)
Supplement: Supplementary file 1 — Supplementary Material 1 [file 41598_2026_45618_MOESM1_ESM.pdf]

**Supplementary Information**

**Title:** A Novel Prediction Model Based on an Adaptive Network Neutrosophic Hesitant Fuzzy Inference System (ANNHFIS): A Case Study of Istanbul

**Authors:** Ataullah Turgut & Sukran Seker

**Supplementary Table S1.** Results of data homogeneity and trend tests.

| Data Type | Data Name | SNHT | Mann–Kendall Tests |
|---|---|---|---|
| Training | Solar Radiation ($W/m^2$) | Not Normally Distributed | No Trend |
| Training | Air Temperature (°C) | Not Normally Distributed | No Trend |
| Training | $NO_x$ ($\mu g/m^3$) | Not Normally Distributed | No Trend |
| Training | Relative Humidity (%) | Not Normally Distributed | No Trend |
| Training | $PM_{10}$ ($\mu g/m^3$) | Not Normally Distributed | No Trend |
| Validation | Solar Radiation ($W/m^2$) | Not Normally Distributed | Trend |
| Validation | Air Temperature (°C) | Not Normally Distributed | Trend |
| Validation | $NO_x$ ($\mu g/m^3$) | Not Normally Distributed | Trend |
| Validation | Relative Humidity (%) | Not Normally Distributed | Trend |
| Validation | $PM_{10}$ ($\mu g/m^3$) | Not Normally Distributed | Trend |
| Testing | Solar Radiation ($W/m^2$) | Not Normally Distributed | Trend |
| Testing | Air Temperature (°C) | Not Normally Distributed | No Trend |
| Testing | $NO_x$ ($\mu g/m^3$) | Not Normally Distributed | No Trend |
| Testing | Relative Humidity (%) | Not Normally Distributed | No Trend |
| Testing | $PM_{10}$ ($\mu g/m^3$) | Not Normally Distributed | Trend |

**Supplementary Table S2.** Parameter optimization settings for all compared models.

| **Method** | **Parameter Optimization** | **Optimum Parameter** | **Controlled Ranges** |
|---|---|---|---|
| ANNHFIS | PSO | - | Mean parameters: [0.0, 1.1]<br>Sigma parameters: [0.001, 0.5] |
| ANNHFIS | GS | Delta: 0.05,<br>Sigma scaling: 1.0 | Delta: [0.05, 0.1, 0.15],<br>Sigma scaling: [0.8, 0.9, 1.0, 1.1, 1.2] |
| ANFIS | PSO | - | Mean parameters: [0.0, 1.1]<br>Sigma parameters: [0.001, 0.5] |
| ANFIS | GS | Delta: 0.05,<br>Sigma scaling: 0.8 | Delta: [0.05, 0.1, 0.15],<br>Sigma scaling: [0.8, 0.9, 1.0, 1.1, 1.2] |
| MLP-ANN | GS | hidden_layer=(200, 100),<br>activation=tanh,<br>solver=adam | hidden_layer_sizes: [(100, 50), (200, 100)],<br>activation: [relu, tanh],<br>solver:[adam, sgd] |
| LSTM | GS | units=50,<br>num_layers=2,<br>batch_size=16,<br>epochs=50 | units ∈ [25, 32, 40, 50, 64],<br>num_layers ∈ [1, 2],<br>batch_size ∈ [8, 16, 32, 64],<br>epochs ∈ [50, 60, 100, 150, 200] |

**Supplementary Table S3.** Hyperparameter settings and selected values (by method).

| Method | Search/optimizer | Search space (key hyperparameters) | Training/optimization settings | Final selection (if specified) |
|---|---|---|---|---|
| ANNHFIS-PSO | PSO + fine-tuning | PSO: particles=50, iters=50, pso_epochs=100; INITIAL_ETA=3e-4; λ_reg=0.1 | w=0.79, c1=1.77, c2=1.53; fine-tune epochs=120 | Best particle vector + fine-tune |
| ANNHFIS-GS | Full grid over NHFS params | Δ ∈ {0.05, 0.1, 0.15}, σ_factor ∈ {0.8–1.2}, η ∈ {1e-5–1e-3}, λ_reg, λ_LSE ∈ {1e-1–1e-3} | TRAIN_EPOCHS=500, lr reduction, early stopping (patience=10) | Best by validation $R^2$ |
| ANFIS-PSO | PSO + hybrid update | PSO: particles=50, iters=50, pso_epochs=150; MF means [0, 1.1], sigmas [0.001, 0.5] | w=0.6, c1=1.7, c2=1.8; fine-tune epochs=50 | Best particle by objective |
| ANFIS-GS | Full grid over MF placements | Two Gaussian MFs per input; Δ ∈ {0.05, 0.1, 0.15}, σ_factor ∈ {0.8, 0.9, 1.0, 1.1, 1.2} | Hybrid training up to 100 epochs, early stopping; lr=0.01, patience=10 | Best by validation $R^2$ |
| MLP-ANN | GS | hidden_layer_sizes ∈ {(100, 50), (200, 100)}, activation ∈ {relu, tanh}, solver ∈ {adam, sgd} | max_iter=1000, random_state=42 | hidden_layer=(200, 100), activation=tanh, solver=adam |
| LSTM | GS + fine-tune | units ∈ {25–64}, layers ∈ {1, 2}, batch ∈ {8–64}, epochs ∈ {50–200} | Adam optimizer, lr=1e-3/1e-4, up to 200 epochs | units=50, layers=2, batch=16, epochs=50 |
