# Supplementary material for: A novel nowcasting (estimation) model based on an adaptive network neutrosophic hesitant fuzzy inference system (ANNHFIS): a case study of Istanbul
Source: Sci Rep. 2026 Mar 24;16:14855. doi: 10.1038/s41598-026-45618-7 (PMC13168669; doi:10.1038/s41598-026-45618-7)
Supplement: Supplementary file 2 — Supplementary Material 2 [file 41598_2026_45618_MOESM2_ESM.docx]

**Supplementary Information**

**Title:** A Novel Nowcasting (Estimation) Model Based on an Adaptive Network Neutrosophic Hesitant Fuzzy Inference System (ANNHFIS): A Case Study of Istanbul

**Authors:** Ataullah Turgut & Sukran Seker

**Supplementary Table S1.** Results of data homogeneity and trend tests.

| Data Type | | Data Name | SNHT | Mann–Kendall Tests |
| --- | --- | --- | --- | --- |
| Training | Solar Radiation (W/m²) | | Not Normally Distributed | No Trend |
| Training | Air Temperature (°C) | | Not Normally Distributed | No Trend |
| Training | NOₓ (μg/m³) | | Not Normally Distributed | No Trend |
| Training | Relative Humidity (%) | | Not Normally Distributed | No Trend |
| Training | PM₁₀ (μg/m³) | | Not Normally Distributed | No Trend |
| Validation | Solar Radiation (W/m²) | | Not Normally Distributed | Trend |
| Validation | Air Temperature (°C) | | Not Normally Distributed | Trend |
| Validation | NOₓ (μg/m³) | | Not Normally Distributed | Trend |
| Validation | Relative Humidity (%) | | Not Normally Distributed | Trend |
| Validation | PM₁₀ (μg/m³) | | Not Normally Distributed | Trend |
| Testing | Solar Radiation (W/m²) | | Not Normally Distributed | Trend |
| Testing | Air Temperature (°C) | | Not Normally Distributed | No Trend |
| Testing | NOₓ (μg/m³) | | Not Normally Distributed | No Trend |
| Testing | Relative Humidity (%) | | Not Normally Distributed | No Trend |
| Testing | PM₁₀ (μg/m³) | | Not Normally Distributed | Trend |

**Supplementary Table S2.** Hyperparameter settings and selected values (by method).

| Method | Search/optimizer | Search space & Control Ranges | Training/optimization settings | Final selection (if specified) | Runtime (Minute) |
| --- | --- | --- | --- | --- | --- |
| ANNHFIS-PSO | PSO + fine-tuning | MF means ∈ [0.0, 1.1], MF sigmas ∈ [0.03, 0.5];  PSO: particles=50,  iters=50,  pso_epochs=100; INITIAL_ETA=3e-4;  λ_reg=0.1,  λ__LSE_=1.0,  λ__pen_=0.1 | w=0.79, c1=1.77, c2=1.53, Hybrid learning (Full-batch, LSE + Adam-based GD), Fine-tune: 120 epochs, early stopping (patience=3). PSO: particle-eval early stopping (patience=5) + outer-loop early stopping (PSO_PATIENCE=5). Output clipped to [0,1]. | Optimized MF parameters (Mean/Sigma for T/I/F) & Rule Weights + 120 epochs fine-tuning | 44.4 |
| ANNHFIS-GS | Full grid over NHFS params | Δ ∈ {0.05, 0.1, 0.15},  σ_factor ∈ {0.8, 0.9, 1.0, 1.1, 1.2} η ∈ {1e-5–1e-3},  λ_reg, λ_LSE ∈ {1e-1–1e-3} | TRAIN_EPOCHS=500, Full-batch, lr reduction, early stopping (patience=10) | Δ=0.05, σ_factor =1.0,  η = 1e-4,  λ_reg= 1e-3, λ_LSE = 1e-2 | 31.93 |
| ANFIS-PSO | PSO + Hybrid learning (LSE + GD) | PSO: particles=50,  iters=50,  pso_epochs=150,  MF means [0, 1.1],  sigmas [0.03, 0.5],  λ__LSE_=0.1,  λ__reg_=0.05,  Initial η =5e-5 | w=0.6, c1=1.7, c2=1.8; Hybrid learning (Full-batch, LSE + Adam-based GD), fine-tune epochs=50 (early stopping, patience=3), PSO patience=5 | Optimized MF parameters (Mean/Sigma) + 50 epochs fine-tuning | 2.2 |
| ANFIS-GS | Full grid over MF placements | Δ ∈ {0.05, 0.1, 0.15},  σ_factor ∈ {0.8, 0.9, 1.0, 1.1, 1.2} | Hybrid training up to 100 epochs, early stopping (patience=10, min_delta=1e-6); lr=0.01, λ__reg_=1e-4, | Δ=0.15, σ_factor =1.0, | 7.2 |
| MLP-ANN | GS | hidden_layer_sizes ∈ {(100, 50), (200, 100)},  activation ∈ {relu, tanh},  solver ∈ {adam, sgd} | max_iter=1000, random_state=42,  batch_size='auto', early_stopping=False; alpha=1e-4, tol=1e-4 | hidden_layer=(200, 100), activation=tanh, solver=adam | 0.21 |
| LSTM | GS + fine-tune | units ∈ {25, 32, 40, 50, 64},  layers ∈ {1, 2},  batch ∈ {8, 16, 32, 64},  epochs ∈ {50, 60, 100, 150, 200} | Adam (lr=1e-3),  Loss=MSE, No dropout,  Fixed epochs (no early stopping) | units=50, layers=2, batch=16, epochs=50 | 120.38 |
